# Diagnostic performance of coronary artery disease prediction model (CAD-score system) based on phonocardiogram: a systematic review and meta-analysis

**DOI:** 10.3389/fcvm.2025.1575230

**Published:** 2025-11-05

**Authors:** Miftah Pramudyo, Ahmad Faried, Mohammad Rizki Akbar, Dzulfikar Djalil Lukmanaul Hakim, Agung Wahyu Setiawan, William Kamarullah

**Affiliations:** ^1^ Department of Cardiology and Vascular Medicine, Faculty of Medicine, Universitas Padjadjaran, Bandung, Indonesia; ^2^ Department of Neurosurgery, Faculty of Medicine, Universitas Padjadjaran – RS Unpad University Hospital, Jatinangor, Indonesia; ^3^ Doctoral Program, Universitas Padjadjaran, Bandung, Indonesia; ^4^ Department of Child Health, Faculty of Medicine, Universitas Padjadjaran, Bandung, Indonesia; ^5^ School of Electrical Engineering and Informatics, Institut Teknologi Bandung, Bandung, Indonesia

**Keywords:** coronary artery disease, chronic coronary syndrome, phonocardiography, heartsound, pre-test probability

## Abstract

**Introduction:**

Pre-test probability stratification of individuals with suspected obstructive coronary artery disease (CAD) has remained suboptimal for many years. Consequently, the majority of diagnostic tests used to rule out CAD exhibit normal results. An acoustic device capable of measuring micro bruits caused by stenosis-induced turbulence in the coronary circulation has showcased potential for stratifying CAD. The aim of this meta-analysis was to investigate the conceivable diagnostic value of phonocardiogram (PCG) in detecting the presence of CAD.

**Methods:**

We conducted a comprehensive search of PubMed, EuropePMC, and ScienceDirect for articles published through January 2025. Studies were eligible if they assessed the accuracy of PCG using the CADScor® system in predicting CAD and provided enough data to construct a 2 × 2 contingency table.

**Results:**

A total of 4 studies involving 4,050 patients were included for the final analysis. The pooled sensitivity and specificity were 87% (95% CI, 80%–92%) and 35% (95% CI, 21%–52%), respectively. The pooled positive likelihood ratio (PLR) was 1.34 (95% CI, 1.09–1.64) and the pooled negative likelihood ratio (NLR) was 0.37 (95% CI, 0.25–0.55). The area under the receiver operating characteristic curve (AUC) was 0.79 (95% CI, 0.75–0.82) in predicting CAD. Fagan's nomogram showed that the posterior probability of PCG with the CADScor® system for the detection of CAD was 19% when the CAD-score was above the cut-off value, and 6% in those with CAD-score below the cut-off value.

**Conclusion:**

Phonocardiography shows promise as a rule-out tool for patients with suspected CAD.

**Systematic Review Registration:**

https://www.crd.york.ac.uk/PROSPERO/view/CRD42024550526, PROSPERO CRD42024550526.

## 1 Introduction

Coronary artery disease (CAD) has been identified as a frequent yet highly perilous cardiovascular condition, incorporating one of the major causes of hospital admission among adults globally, with over 1 million hospitalizations each year (1). Unlike acute coronary syndrome, stable CAD (or currently known as chronic coronary syndrome) advances slowly and chronically and often yields atypical symptoms, rendering it a tough row to hoe to determine whether the patient requires further non-invasive testing as an initial work-up to rule out this malady, which is a major concern for most of emergency healthcare providers (2, 3).

Current risk stratification measures, such as the Diamond-Forrester score (DF-score), which is used to stratify people with chest pain and possible stable CAD, have been proven to greatly overestimate the disease's likelihood. Several recent investigations have demonstrated that merely 6%–12% of those referred for non-invasive testing suffer from significant CAD. This implies that the remaining 88%–94% of patients with normal coronary arteries (NCA) had to undergo an array of expensive, time-consuming, hazardous, and futile examinations (4, 5). Hence, there is growing concern about the need for practical, swift, and cost-effective diagnostic strategies, particularly for discerning obstructive CAD.

The notion of analyzing heart sounds using spectrum analysis was initially used in the 1950s to identify various cardiac diseases, including valvular heart disease and CAD (6, 7). Several investigations have found that coronary artery blockages can cause high-frequency murmur sounds arising from turbulent blood flow colliding with partially occluded arteries. The murmurs are beyond mere turbulence; they comprise an intricate combination of signals caused by vibrations within the surrounding cardiac walls, producing noises that are difficult to perceive with the human ear (8–12). Nonetheless, current state-of-the-art computer and acoustic technology advancements have resulted in identifying heart sounds more effortlessly, which facilitates the prompt risk assessment of CAD (13, 14).

Phonocardiogram (PCG), a non-invasive, yet low-cost modality, is an option for diagnosing CAD by utilizing heart sounds. PCG systems such as the CAD-score analyze heart sounds, particularly during diastole for patterns of turbulence caused by coronary artery narrowing. The system derives acoustic features including spectral slope, signal entropy, and heart rate variability, which are then combined with clinical variables (e.g., age, sex, blood pressure) to generate a risk score from 0 to 99 (15). The correct categorization of patients at risk of CAD using this PCG tool, coupled with the existing risk stratification scoring system, may not only lower health expenses, but also drawbacks from non-invasive and invasive procedures. However, data on its efficacy and accuracy remains scarce due to the small-scale number of investigations and diverse algorithms approach throughout available studies, therefore lack of accuracy. Our aim was to yield more robust evidence by pooling all these data in this meta-analysis in order to achieve better identification and reliability in diagnosing stable CAD.

## 2 Materials and methods

### 2.1 Protocol and registration

This meta-analysis was conducted following the Preferred Reporting Items for Systematic Reviews and Meta-analyses (PRISMA) recommendations. The review protocol was registered in the PROSPERO (Prospective Register of Systematic Reviews) international registry database under registration number CRD42024550526 (16).

### 2.2 Search strategy

**Figure 1:**
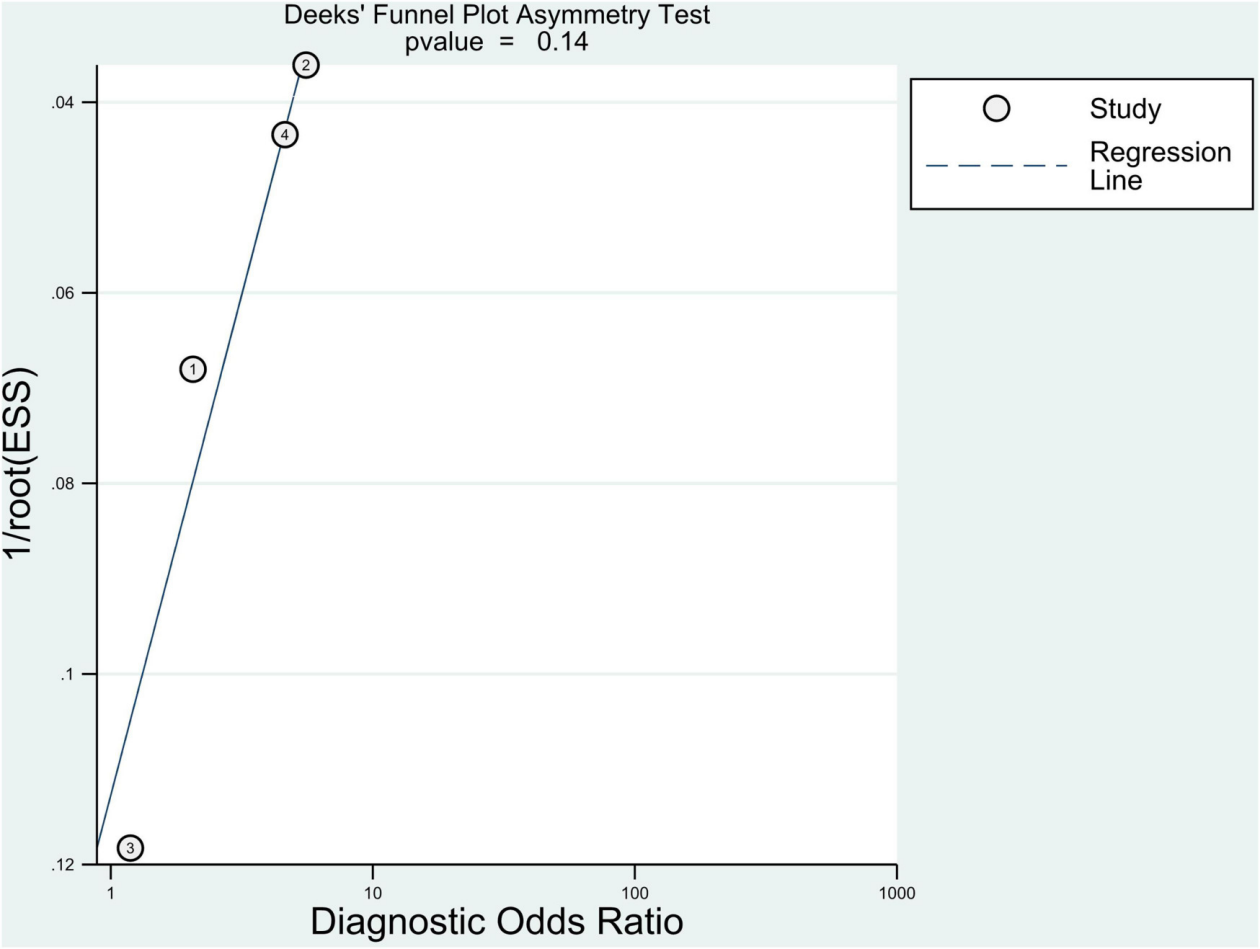
PRISMA diagram of selection process for studies included in the meta-analysis.

We reviewed the databases PubMed, Europe PMC, and ScienceDirect up to June 2025. The search terms were as follows: (“phonocardiogram” OR “phonocardiography” OR “heart sound” OR “murmur” OR “cardiac auscultation”) AND (“coronary artery disease” OR “chronic coronary syndrome” OR “angina” OR “stable ischemic heart disease”). When required, the reference lists of the included research and relevant review papers were scrutinized for additional references. We adapted the search terms to fit the particular requirements of each database. Our search as in line with PRISMA principles, and the flowchart in Figure 1 portrays the search and screening procedures.

### 2.3 Study selection and eligibility criteria

We included both prospective and retrospective observational studies reporting the predictive value of PCG in diagnosing CAD (categorical) compared with invasive coronary angiography (ICA) in this meta-analysis. The detailed inclusion criteria were as follows: (1) CAD patients over 18 years old, characterized by symptoms suggestive of stable angina pectoris; (2) studies reported key exposure between high- and low-PCG score, defined as a value surpassing cut-off point determined within each study in a comparative manner between the aforementioned variable. Our investigation required studies to publish sufficient information to generate a 2 × 2 table consisting of true positive, false positive, false negative, and true negative to compute the effect size.

We excluded studies in which patients: (1) presented with acute coronary syndrome; (2) valvular heart disease and other pre-existing diagnosis which could generate cardiac murmurs and affect the quality of auscultation; (3) previous coronary revascularization (either percutaneous coronary intervention or coronary artery bypass graft); (4) any contraindications to ICA; (5) any arrhythmias that caused non-sinus rhythm; (6) substantial skin impairments or any aberrant anatomies at the site of auscultation; (7) pregnancy. Our analysis omitted studies that failed to provide sufficient of the aforementioned data. Animal studies, review papers, editorials, comments, letters to editors, case reports/series, meta-analyses, and conference abstracts were also disregarded from our meta-analysis.

### 2.4 Phonocardiogram vs. control groups

Due to the vast number of articles employing multiple distinct PCG analysis methods, our research included studies utilizing the CADScor® System, Acarix A/S, Kongens Lyngby, Denmark, since only investigations involving this sort of PCG could be merged in the meta-analysis. Following a 5-min rest interval, the patient's heart sounds were collected in the supine position for 3 min utilizing the aforementioned device in the left fourth intercostal region, with four bouts of eight-second breath holding. The aim of holding breaths while recording was to avert breathing noise, which could obscure heart sounds. Audio recording was also carried out in a quiet environment. To ensure the blinding protocol, the acoustic analysis findings were not directly presented on the acoustic detection system. The CAD-Score System's final score was derived using an integrated pre-existing algorithm that included eight acoustic features as well as combining numerous factors such as age, gender, and blood pressure information. If the recording equipment detected poor sound quality, it would instantaneously request a re-recording. The CAD-score varied from 0 to 99. After the recording was completed, a CAD-score of more than 20 was classified as CAD; otherwise, it was classified as non-CAD (9, 10, 12).

The reference standard for diagnosing or ruling out obstructive CAD is ICA. Two cardiologists will blindly evaluate the coronary angiography (CAG) results independently to determine the degree of vascular stenosis. Patients with stenosis of at least 50% of the diameter were classified as significant CAD, while the remainder were classified as non-CAD (9, 10, 12). Our research protocol allowed for investigations encompassing computed tomography coronary angiography (CTCA) as a supplemental examination, to be employed as additional data analysis for determining the diagnostic accuracy of PCG.

### 2.5 Data extraction and risk of bias assessment

The course of data abstraction was conducted by two authors independently using a form detailing baseline characteristics of the included studies, such as first author's name, age, male sex, study design, the country in which the study was conducted, ethnicity, chest pain characteristics, body mass index (BMI), hypertension, diabetes mellitus (DM), smoking status, hypercholesterolemia, Diamond-Forrester pre-test probability (PTP) score, CTCA characteristics, ICA characteristics, and follow-up length. If the study did not disclose mean and standard deviation, an estimate was utilized using a method proposed by Wan et al. (17). Two independent reviewers managed the whole research selection and data extraction process. Disagreements over study selection and data extraction were handled by consensus or a third reviewer.

The Newcastle-Ottawa Scale (NOS) was implemented by the authors to independently assess the possibility of bias in each study. A study with a total score of seven or above was deemed bias-free. Research with a total score of six or less was considered to be biased and thus excluded from the research. Author discussion was applied to settle quality rating disagreements (18). Discrepancies in quality ratings were resolved through discussion with a third reviewer.

### 2.6 Statistical analysis

In this meta-analysis, we implemented STATA 17.0 to calculate the overall effect magnitude. A bivariate random-effects regression model was performed to determine sensitivity, specificity, positive likelihood ratio (PLR), and negative likelihood ratio (NLR). A summary receiver operating characteristic (SROC) curve was drawn to assess the overall diagnostic accuracy. The higher the area under the curve (AUC), the greater the diagnostic power. Fagan's nomogram was plotted to assess the connection between prior and post-test probability. Deek's funnel plot was used to identify any publication bias. A *P*-value less than 0.10 denotes a publication bias. Univariable meta-regression and subgroup analyses for the output of diagnostic test accuracy meta-analysis with possible covariates were also performed if the included studies were sufficient to generate the results. All statistical tests were two-sided, and *P* < 0.05 indicated statistical significance.

## 3 Results

### 3.1 Study selection and characteristics of the included studies

Figure 1 depicts the results of the literature search. After identifying duplicates, we discovered 472 pre-screened articles, reviewed the title and abstract of the remaining articles, and omitted 428 records. The remaining 44 entries' full-text articles were obtained and investigated. Eventually, 40 studies were deemed ineligible, and the remaining 4 were chosen for qualitative and quantitative analyses. The mean age of the participants was 60.3 ± 3.3 years, 51.2% of patients were male, with a total of 4,050 participants. Previous hypertension, DM, smoking status, and hypercholesterolemia were present in 65.9%, 15.9%, 24.7%, and 54.2% of patients, respectively. 55.7% of patients presented with typical chest pain. The cut-off value for CAD-score was set at 20 amidst the studies.

Baseline and clinical characteristics of the included studies were listed in Table 1. Calculations from PTP were made using three pre-defined probability categories based on DF-score: low risk: 10.2%; intermediate risk: 82.3%; high risk: 7.5%. Of the patients referred for ICA, 15.8% were later classified as obstructive CAD (≥50% stenosis). Based on the results of the CTCA, patients were grouped into non-CAD (42.2%), mild CAD (29.4%), moderate CAD (8,9%), and severe CAD (19.5%). The mean CAD-score calculated from spectrum analysis was 25.8 ± 4.6 in the non-CAD group, which was significantly lower than the CAD-score in the significant CAD group (38.5 ± 6.2).

**Table 1:** Baseline characteristics of the included studies.

No.	Author (year)	Country	Study design	Sample size (n)	PCG characteristics	Mean age (years)	Male (%)	Ethnicity (%)	Chest pain characteristics (%)	BMI (kg/m^2^)	Hypertension (%)	Blood pressure (mmHg)	DM (%)	Smoking (%)	Hypercholesterolemia (%)	Diamond Forrester PTP score	CTCA characteristics	ICA characteristics	NOS
1	Renker et al. (9)	Germany	POS	226	CADScor® System, Acarix A/S, Kongens Lyngby, Denmark Mean CAD score: Non-CAD: 31.5 ± 13.2 CAD: 41.2 ± 11.9	64.6 ± 10.6	60.2	Caucasian: 99.6	Typical: 85.6 Atypical: 10.6 Non-specific: 3.5	28.2 ± 5.32	85	SBP: 135 ± 17.7 DBP: 77.1 ± 13.6	27.9	44.2	NR	Mean score: NR Low risk (<15%): 0% Moderate risk (15–85%): 82.3% High risk (>85%): 17.7%	N/A	≥50% stenosis: 39.3% 1VD: 23% 2VD: 9.7% 3VD or LMD: 6.6%	9
2	Schmidt et al. (10)	Denmark	ROS	2,245	CADScor® System, Acarix A/S, Kongens Lyngby, Denmark Mean CAD score: Non-CAD: 26.4 ± 14.3 CAD: 38.4 ± 13.9	58.3 ± 8.4	47.2	NR	Typical: 21.8 Atypical: 27.1 Non-specific: 51.1	26.9 ± 4.2	58.5	NR	5.3	16	76	Mean score: 36.4 ± 21.2% Low risk (<15%): 16.5% Moderate risk (15–85%): 81.2% High risk (>85%): 2.3%	CACS score: 0: 48.8% 1–399: 41.3% ≥400: 9.6% Non-CAD: 36.6% Mild-moderate: 20.1% Severe: 15.2%	≥50% stenosis: 9.4%	8
3	Schnaubelt et al. (11)	Austria	POS	105	CADScor® System, Acarix A/S, Kongens Lyngby, Denmark Mean CAD score: Non-CAD: 25 ± 15.9 CAD: 44.3 ± 17.1	61 ± 15.1	48.6	Caucasian: 94.3	Typical: 87.6 Atypical: 5.7 Non-specific: 6.7	28 ± 6.1	54.3	SBP: 142.7 ± 19.5 DBP: 78 ± 15.1	24.8	22.9	32.4	NR	N/A	≥50% stenosis: 3.8%	8
4	Winther et al. (12)	Denmark	POS	1,474	CADScor® System, Acarix A/S, Kongens Lyngby, Denmark Mean CAD score: Non-CAD: 20.4 ± 12 CAD: 29.9 ± 12	57.2 ± 8.8	48.8	Caucasian: 99.3	Typical: 27.8 Atypical: 33.6 Non-specific: 38.6	26.7 ± 4.1	NR	SBP: 138 ± 19 DBP: 83 ± 11	5.4	15.8	NR	Mean score: 37.7 ± 25.2% Low risk (<15%): 14.2% Moderate risk (15–85%): 83.4% High risk (>85%): 2.4%	CACS score: 0: 51.1% 1–399: 38.5% ≥400: 10.4% Non-CAD: 47.8% Mild: 20.8% Moderate: 7.6% Severe: 23.8%	≥50% stenosis: 10.6% 1VD: 6.5% 2VD: 2.9% 3VD or LMD: 1.2%	9

1VD, one-vessel disease; 2VD, two-vessel disease; 3VD, three-vessel disease; BMI, body mass index; CACS, coronary artery calcium score; CAD, coronary artery disease; CTCA, computed tomography coronary angiography; DBP, diastolic blood pressure; DM, diabetes mellitus; ICA, invasive coronary angiography; kg/m2, kg/m2, kilograms divided by the square of the height in meters; LMD, left main disease; mmHg, millimeter of mercury; N/A, not available; NOS, Newcastle-Ottawa Scale; NR, not reported; PCG, phonocardiogram; POS, prospective observational study; PTP, pre-test probability; ROS, retrospective observational study; SBP, systolic blood pressure.

### 3.2 Diagnostic-test accuracy meta-analysis

**Figure 2:**
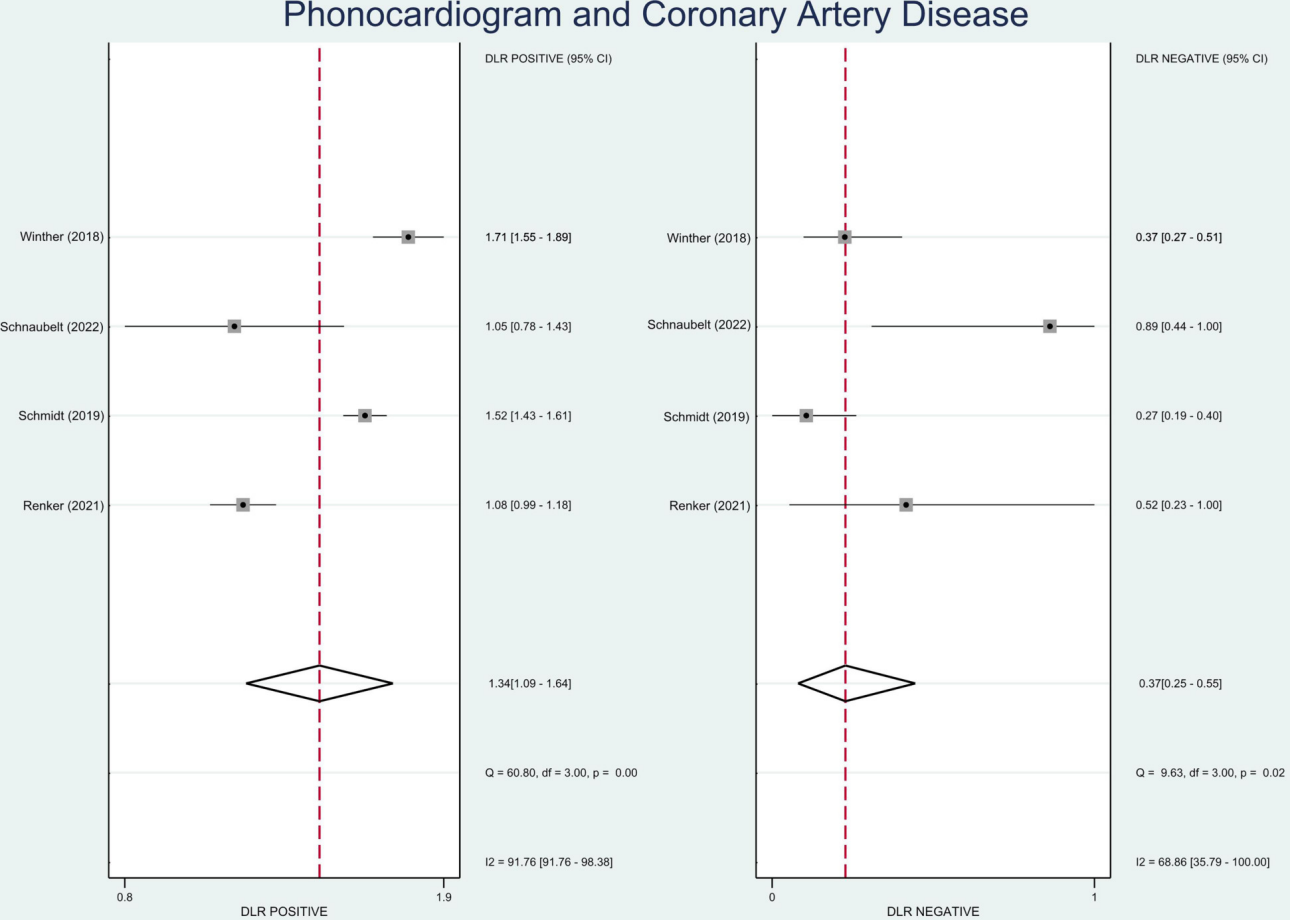
Forest plot showing pooled sensitivity and specificity of PCG in diagnosing CAD.

**Figure 3:**
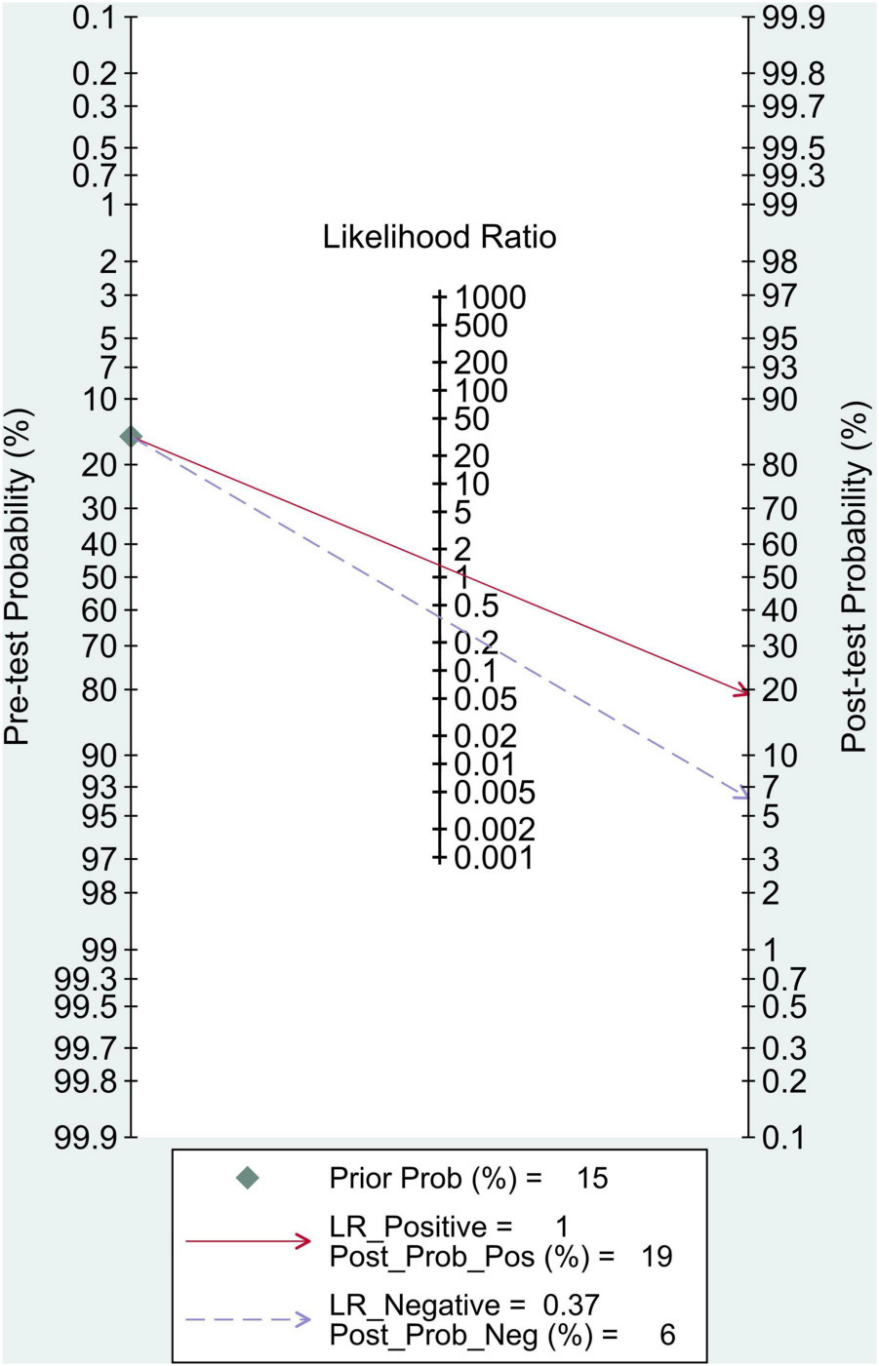
Forest plot showing positive and negative likelihood ratio of PCG in diagnosing CAD.

**Figure 4:**
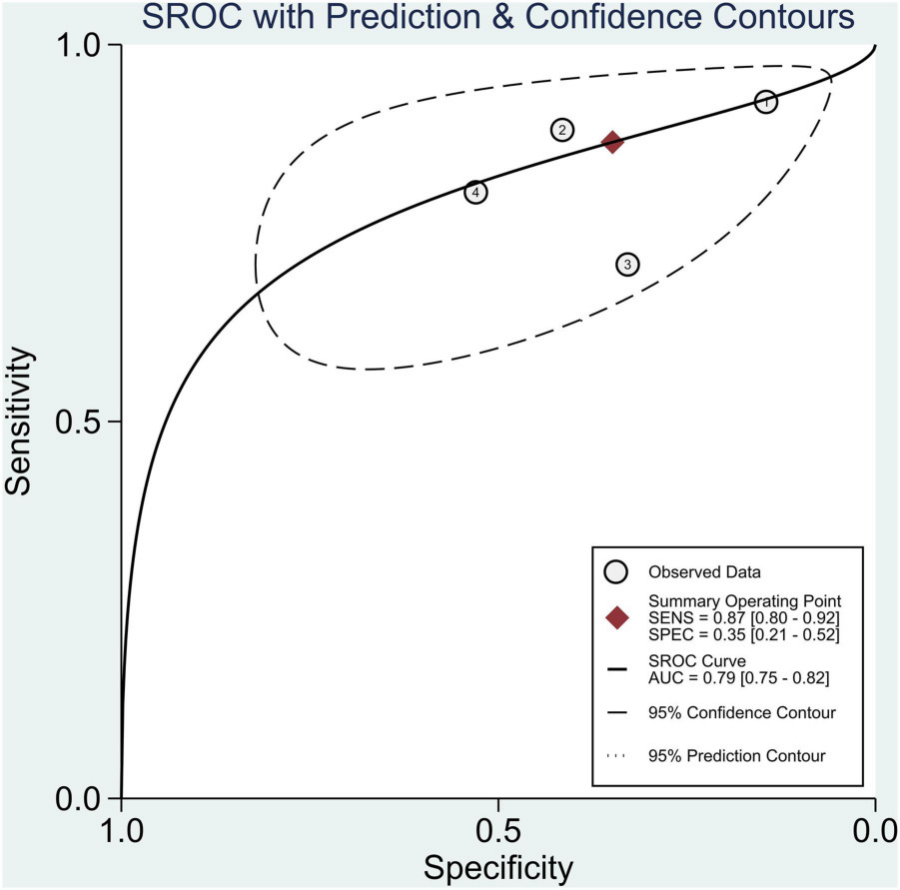
Summary of receiver operating characteristic plots for diagnostic accuracy of PCG in diagnosing CAD.

**Figure 5:**
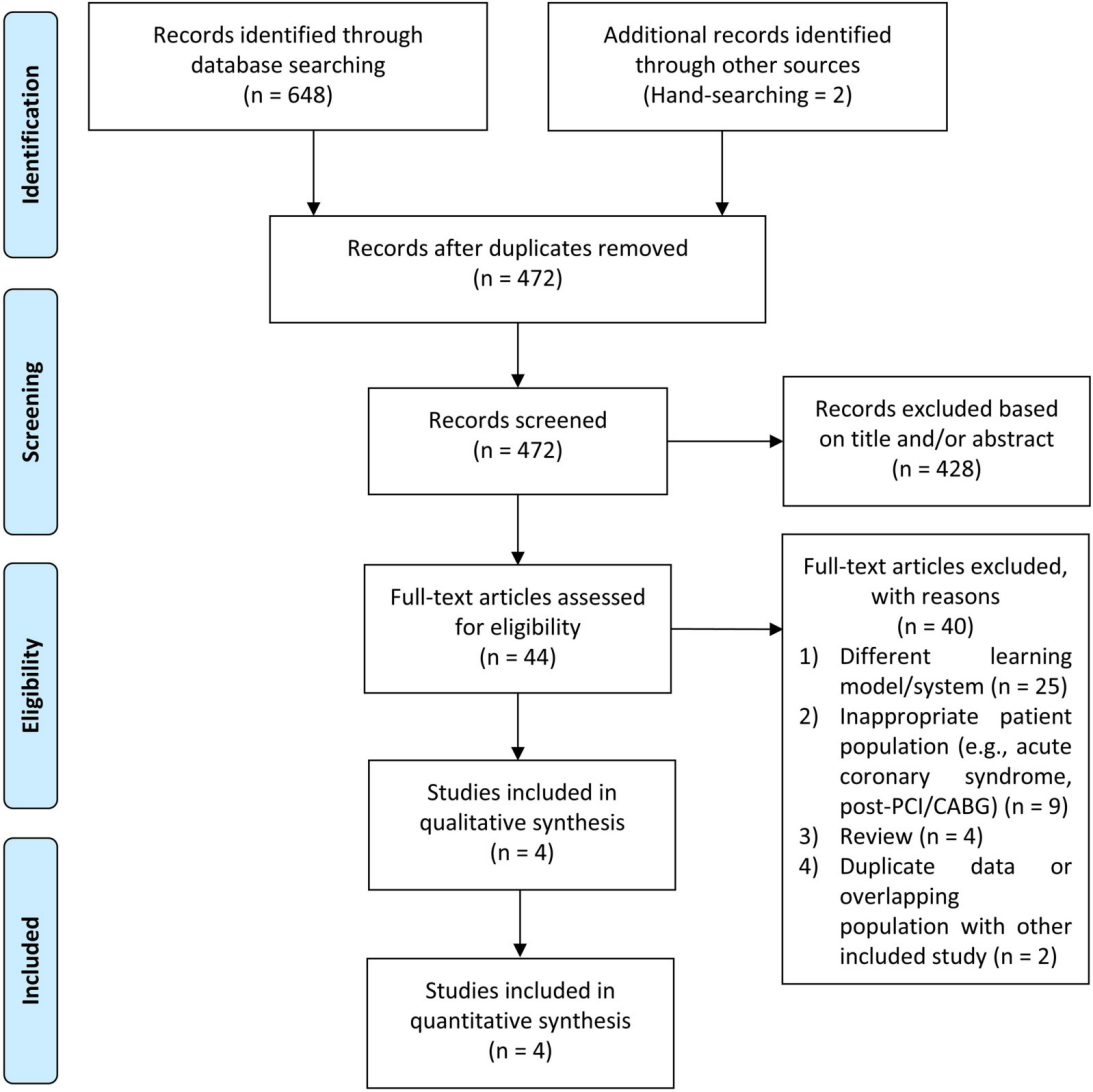
Fagan's nomogram.

The pooled sensitivity and specificity were 87% (95% CI, 80%–92%) and 35% (95% CI, 21%–52%), respectively (Figure 2). The PLR was 1.34 (95% CI, 1.09–1.64), and the NLR was 0.37 (95% CI, 0.25–0.55) (Figure 3). The SROC curve is shown in Figure 4. The AUC of PCG for predicting CAD was 0.79 (95% CI, 0.75–0.82), indicating moderate-high diagnostic value. Fagan's nomogram showed that if the pre-test probability was set to 15%, the post-test probability of PCG for the detection of CAD was 19% when the CAD-score was above the cut-off value. On the contrary, when the CAD-score was below the cut-off value, the post-test probability was 6% (Figure 5).

### 3.3 Publication bias

**Figure 6:**
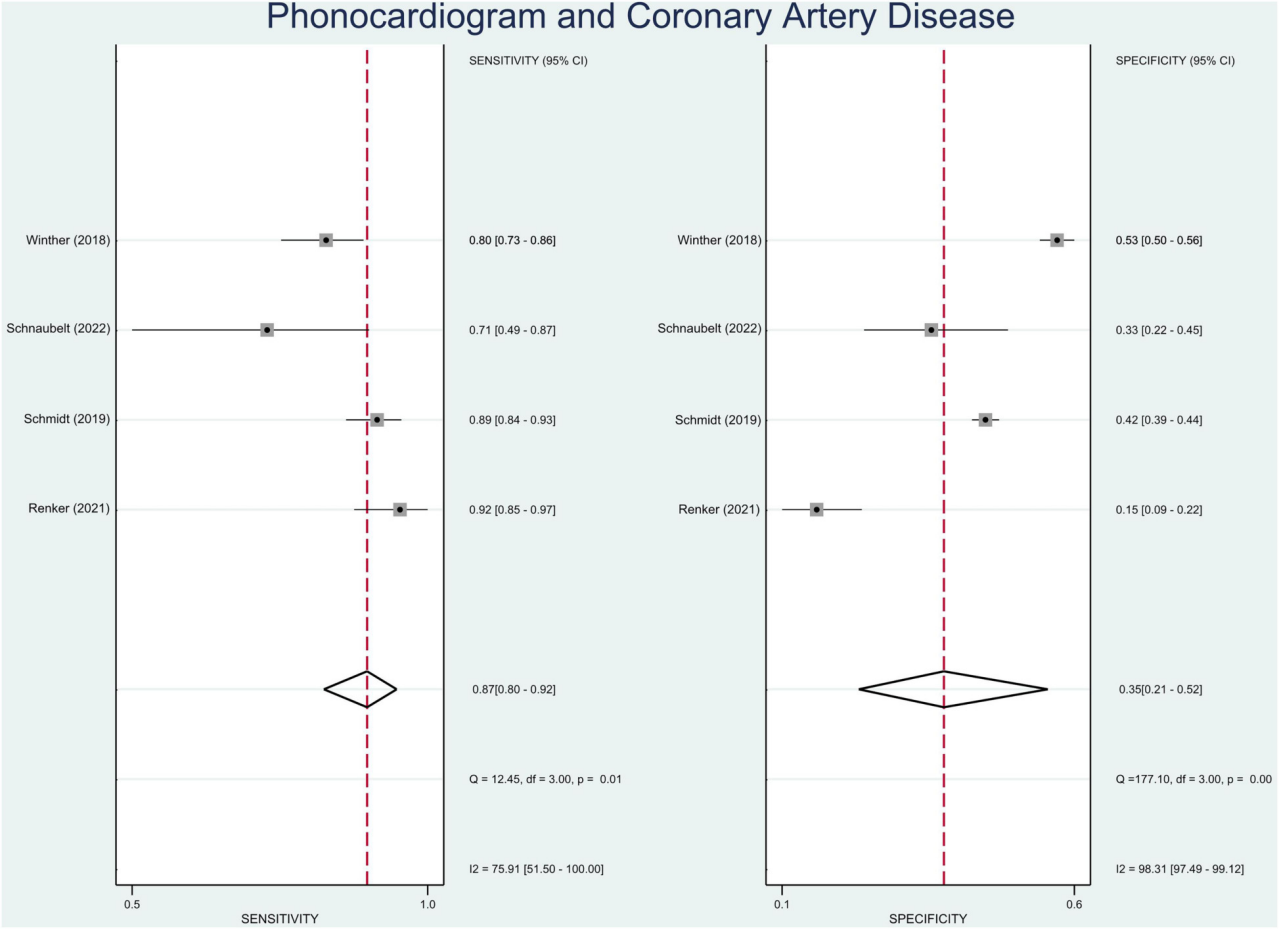
The Deeks’ funnel plot for assessment of potential publication bias of the included studies.

In this meta-analysis, Deek's funnel plot asymmetry test was used to determine the likelihood of publication bias. The Deek's funnel plot was rather symmetrical in comparison to the regression line, and the asymmetry test was non-significant (*P* = 0.14) (Figure 6). The mean NOS of the included studies was 8.5 ± 0.58, indicating a low risk of bias.

## 4 Discussion

Early detection and agile awareness methods for coronary artery disease are crucial issues requiring additional investigation as any late acknowledgments can result in misdiagnosis and futile medical procedures, which may subsequently impair clinical progression, raise the healthcare expenses, and complications related to the testing. The most notable finding in this study was that PCG, specifically the CAD-score, had a considerably high predictive value for identifying CAD, comprising a sensitivity of 87%, specificity of 35%, an AUC value of 0.79, with PLR and NLR of 1.34 and 0.37, respectively. Given its high sensitivity and low negative likelihood ratio, PCG is more suitable as a rule-out rather than a rule-in modality. A negative CAD-score substantially reduces the post-test probability of obstructive CAD, reinforcing its role in excluding disease in low- to intermediate-risk populations. In contrast, the modest increase in post-test probability with a positive CAD-score highlights that PCG should not be used as a confirmatory test but rather as a triage tool that can complement existing risk stratification strategies.

**Figure 7:**
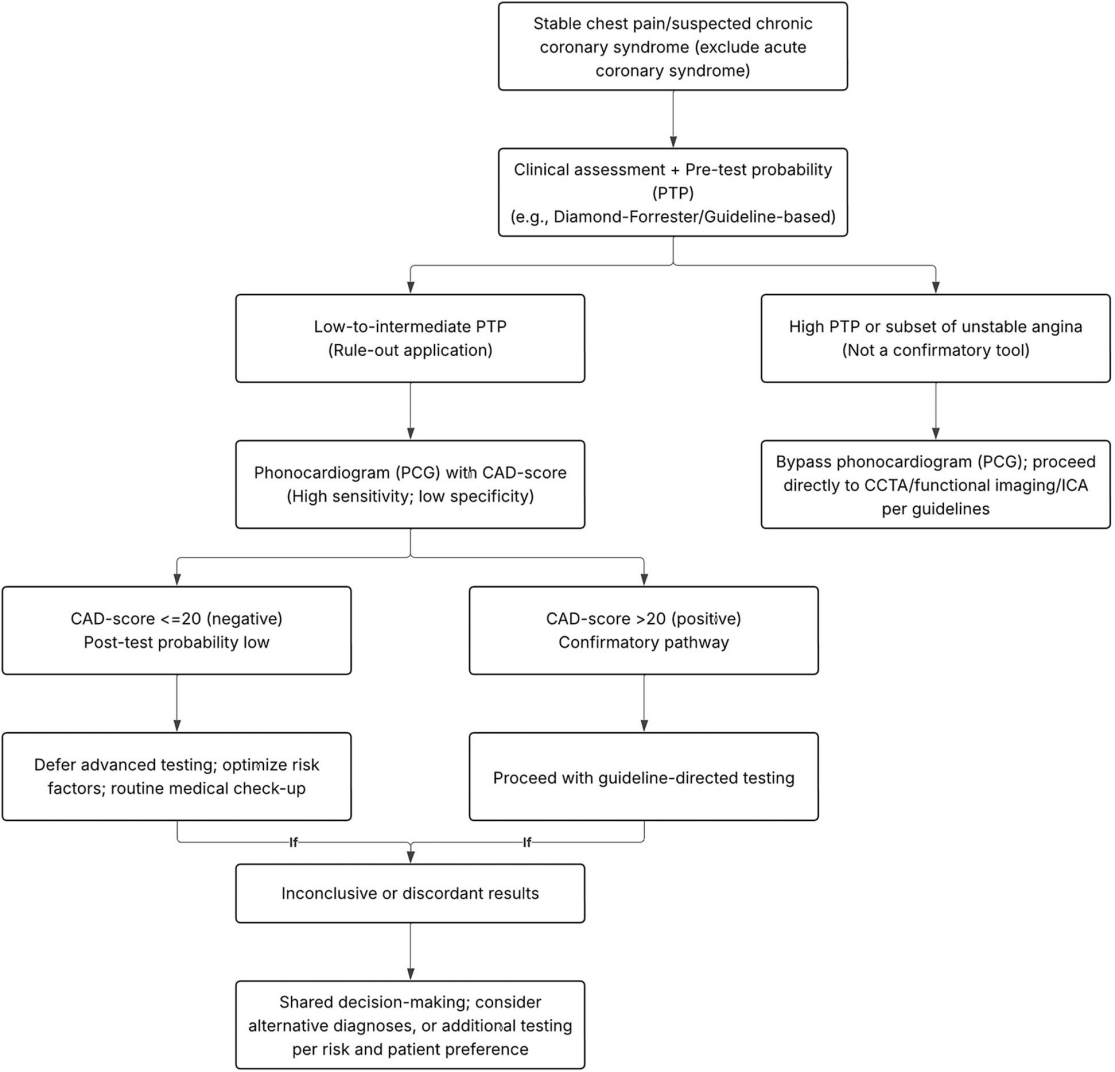
Proposed clinical decision algorithm integrating PCG (CAD-score) into stable chest pain workflows.

Figure 7 outlines a pragmatic pathway in which PCG is used to rule out obstructive CAD among low–intermediate PTP patients; positive results trigger guideline-directed confirmatory testing (CTCA/functional imaging), whereas high-PTP patients bypass PCG. To further illustrate clinical application, we employed Fagan's nomogram to estimate post-test probabilities. When a CAD-score exceeds the cut-off value, the post-test probability rises modestly to 19% from a 15% pre-test estimate, suggesting that a positive PCG result should not be interpreted as confirmatory. In contrast, patients with a CAD-score below the threshold showed a substantial reduction in post-test probability to 6%, reinforcing the CAD-score's value as a rule-out tool. This supports its use in low- to intermediate-risk populations to avoid unnecessary downstream testing. To our knowledge, this is thus far the first meta-analysis evaluating PCG in predicting CAD, as will be captivatingly discussed in the remainder of this article.

To fully comprehend the association between PCG and CAD detection, it is necessary to understand the critical mechanism of PCG, as the findings of this meta-analysis are directly tied to the disease's pathophysiology. Coronary blood flow reaches its highest during the diastole phase (19). Thus, the rise in energy, frequency, and murmur produced during this period is commonly utilized as a feature model to identify coronary artery blockage on a PCG device. The CAD-Score System features an autonomous algorithm that may detect discrepancies in heart sounds, such as micro bruits that result from turbulence in the obstructed coronary circulation. First, the algorithm will filter cardiac sounds and separated them into systolic and diastolic periods before extracting eight acoustic properties that will be utilized to determine whether the patient has CAD. Using a logistic regression approach, the aforementioned recorded acoustic parameters will be incorporated with additional factors consisting of age, gender, and hypertension to formulate the CAD-score (9–12).

Another aspect worth considering is how an amalgamation of the above features might predict the CAD condition. The device's initial acoustic characteristic is heart rate variability (HRV), as one of the early subclinical manifestations of CAD is cardiac autonomic dysfunction, which results in a reduction in HRV (20). Apart from that, the principal component analysis-based measure of randomness (PCAR) component is used to segment cardiac sounds into systolic and diastolic period, while quantify the frequency spectrum into a single measure (13, 21, 22). This is crucial because CAD produces a murmur sound, which increases the diastolic power spectrum (SpectSlope), frequency distribution of S2 owing to vessel-induced murmur vibration (S2freq), as well as enhanced complexity of the sound produced (Sample Entropy). S4amp (fourth sound Amplitude) is related with extra heart sounds near the end of diastole, indicating a reduction in ventricular compliance as a result of CAD injury (13, 23). However, the frequency power ratio (FPR), which measures low frequency power in the mid diastolic phase, remains disputed in terms of mechanism. This might be produced by the cardiac murmur sound, which can theoretically vary from low to high frequencies (where the murmurs are frequently detected), instead coincidentally happens at low frequencies, increasing the magnitude of the low-frequency sound. Other measurements, such as SysFPR (systolic FPR), are utilized to evaluate right coronary artery stenosis, considering peak blood flow occurs within this period (24).

The potential role of PCG is not only as a handy tool for determining the PTP from obstructive CAD based solely on heart sound analysis; the study done by Schmidt et al. (10) also successful in reclassifying patients with suspected stable CAD into a low probability group that was previously utilizing DF-score characteristics. Prior to the CAD-Score test, 227 individuals were classified as low risk and 1,395 (83.4%) as intermediate risk. CAD-Score System could reclassify 472 out of 1,395 patients to “low risk”. The overall low risk group was increased from 227 to 699 patients. Expressed in percentages, initially only 13.6% of the total patient population were classified as low risk. Post-test with CAD-scoring, 41.8% were classified as low risk and could be ruled out from further diagnostics. This lowered the number of intermediate-risk individuals from 1,395 to 923, or from 83.4% to 55.2%. Given its high sensitivity and low negative likelihood ratio, PCG is best positioned to rule out obstructive CAD in low- to intermediate-risk populations.

Exploration of heterogeneity should ideally be undertaken using meta-regression and sub-group analysis, although this is implausible due to a paucity of research. Hence, the leave-one-out sensitivity analysis was carried out, and it was discovered that one study (9) was deemed as the primary contributor to the high inter-study heterogeneity. This can be explained by the fact that Renker et al. (9) investigation had the greatest prevalence (39.3%) of significant CAD, which is conceded as substantial determinant of positive (PPV) and negative predictive (NPV) values. The device's rule-out capability would be reduced in this high-prevalence cohort as the prevalence increased, as might certainly the PPV, while the NPV decreased (25). These findings underscore that the clinical applicability of PCG is closely tied to the baseline risk of the population in which it is used. Nonetheless, the result of our meta-analysis was fairly robust in the leave-one-out sensitivity analysis; removal of any single study did not alter the statistical significance of the pooled estimate, indicating that our results were stable and reliable.

Several limitations still warrant consideration in this meta-analysis. First, we were unable to do meta-regression to determine the direct effect on our outcomes of interest due to a paucity of research. Second, the majority of the research employed a CAD-Score System in CAD patients with ≥50% stenosis; thus, the results cannot be generalized to other PCG device. Third, CAD-Score System does not indicate the location of stenosis. However, we saw a correlation of the severity of CAD and the CAD-score. Limitations also include a small number of studies, with the available evidence derives almost exclusively from European cohorts with predominantly Caucasian participants. Hence, results should be extrapolated with caution to patients of various ethnic backgrounds. Finally, beyond technical factors, practical issues such as operator training, proper sensor placement, and environmental noise can affect PCG accuracy, despite built-in noise reduction features.

## 5 Conclusion

PCG derived from heart sounds provides great clinical diagnostic information in patients with symptoms suggestive of CAD and should be implemented in the standard of care. PCG is best positioned as a rule-out tool rather than a confirmatory diagnostic test. Its integration into current clinical pathways may help clinicians identify patients at low risk of obstructive CAD who can safely avoid unnecessary advanced testing, thereby reducing costs and patient burden. Larger, multi-center, and multi-ethnic investigations are needed to confirm its diagnostic accuracy, assess its real-world applicability, and establish its integration into clinical pathways.

## Data Availability

The raw data supporting the conclusions of this article will be made available by the authors, without undue reservation.
